# Survival Trends in Children With Tetralogy of Fallot in Sweden From 1970 to 2017

**DOI:** 10.1001/jamanetworkopen.2023.14504

**Published:** 2023-05-22

**Authors:** Johanna Persson, Albert Gyllencreutz Castellheim, Mikael Dellborg, Kok Wai Giang, Christina Karazisi, Araz Rawshani, Zacharias Mandalenakis

**Affiliations:** 1 Department of Pediatrics, Institute of Clinical Sciences, Sahlgrenska Academy, University of Gothenburg, Gothenburg, Sweden; 2 Department of Pediatrics, Queen Silvia Children Hospital/Sahlgrenska University Hospital, Region Västra Götaland, Gothenburg, Sweden; 3 Department of Anesthesiology and Intensive Care Medicine, Institute of Clinical Sciences, Sahlgrenska Academy, University of Gothenburg, Gothenburg, Sweden; 4 Department of Anesthesiology and Intensive Care Medicine, Queen Silvia Children Hospital/Sahlgrenska University Hospital, Region Västra Götaland, Gothenburg, Sweden; 5 Department of Molecular and Clinical Medicine, Institute of Medicine, Sahlgrenska Academy, University of Gothenburg, Gothenburg, Sweden; 6 Department of Medicine, Geriatrics and Emergency Medicine, Sahlgrenska University Hospital, Region Västra Götaland, Gothenburg, Sweden; 7 Department of Cardiology, Sahlgrenska University Hospital, Region Västra Götaland, Gothenburg, Sweden; 8 Adult Congenital Heart Unit, Department of Medicine, Sahlgrenska University Hospital, Region Västra Götaland, Gothenburg, Sweden

## Abstract

**Importance:**

Mortality in patients with tetralogy of Fallot (TOF) has decreased substantially since the start of surgical correction of this abnormality in the 1950s. However, nationwide data in Sweden comparing survival trends among pediatric patients with TOF with the general population are still limited.

**Objective:**

To study survival trends in pediatric patients with TOF and compare them with matched controls.

**Design, Setting, and Participants:**

A Swedish registry-based, nationwide, matched cohort study was conducted; data were collected from national health registers from January 1, 1970, to December 31, 2017. Patients with a registered diagnosis of TOF as well as controls without TOF matched by birth year and sex were included in the study. Follow-up data were collected from birth to age 18 years, death, or the end of follow-up (December 31, 2017), whichever occurred first. Data analysis was performed from September 10 to December 20, 2022. Survival trends among patients with TOF were compared with matched controls using Cox proportional hazards regression and Kaplan-Meier survival analyses.

**Main Outcomes and Measures:**

All-cause mortality during childhood in patients with TOF and matched controls.

**Results:**

The population included 1848 patients (1064 [57.6%] males; mean [SD] age, 12.4 [6.7] years) with TOF and 16 354 matched controls. The number of patients who underwent congenital cardiac surgery (henceforth, surgery group) was 1527 (897 [58.7%] males). In the whole TOF population from birth until age 18 years, 286 patients (15.5%) died during a mean (SD) follow-up time of 12.4 (6.7) years. In the surgery group, 154 of 1527 patients (10.1%) died during a follow-up time of 13.6 (5.7) years with a mortality risk of 21.9 (95% CI, 16.2-29.7) compared with matched controls. When stratified by birth period, a substantial decrease in the mortality risk was noted in the surgery group, from 40.6 (95% CI, 21.9-75.4) in those born in the 1970s to 11.1 (95% CI, 3.4-36.4) in those born in the 2010s. Survival increased from 68.5% to 96.0%. The risk of mortality for surgery decreased from 0.52 in the 1979s to 0.19 in the 2010s.

**Conclusions and Relevance:**

The findings of this study suggest there has been substantial improvement in survival in children with TOF who underwent surgery from 1970 to 2017. However, the mortality rate is still significantly higher in this group compared with matched controls. Predictors of good and poor outcomes in this group need to be further explored, with the modifiable ones evaluated for further outcome improvements.

## Key Points

**Question:**

Have the survival and risk of mortality for congenital cardiac surgery in children with tetralogy of Fallot (TOF) in Sweden changed during the past 50 years?

**Findings:**

This cohort study including 1848 patients with TOF noted increased survival, from 68.5% to 96.0%, in children born in the 1970s compared with those born in the 2010s. The risk of mortality associated with congenital cardiac surgery decreased substantially but was 11 times higher than the risk in controls.

**Meaning:**

This study found that, despite marked success in increasing survival over the past 50 years, the overall mortality risk for patients with TOF who undergo congenital cardiac surgery appears to remain higher than in individuals without TOF.

## Teaser

This cohort study examines changes in the survival rates of individuals born with tetralogy of Fallot from 1970 to 2017 in Sweden.

## Introduction

Tetralogy of Fallot (TOF) is the most common complex congenital heart disease (CHD) with an incidence rate of approximately 4.2 per 1000 live births per year.^1^ The tetralogy consists of 4 anatomic features: nonrestrictive ventricular septal defect, overriding aorta, right ventricular outflow tract obstruction, and subsequent right ventricular hypertrophy. Currently, the diagnosis of TOF is often made by antenatal fetal ultrasonography; however, the condition may be recognized postnatally or even later in life depending on the severity of the malformations and the clinical presentation.

The clinical features of TOF include progressive cyanosis, hypoxia, and heart murmurs noted on auscultation. Surgical correction of TOF includes closure of the ventricular septal defect and right ventricular outflow tract reconstruction, often with pulmonary valve surgery.^2^ Left uncorrected, TOF will mainly lead to varying degrees of heart failure, dyspnea with cyanosis, and chronic hypoxia due to the right-to-left shunt through the ventricular septal defect. A limited number of patients survive to their middle-aged years without congenital heart surgery (henceforth, surgery).^3,4^

The first corrective surgery of TOF was performed by Lillehei in 1954 using extracorporeal circulation by controlled cross-circulation (oxygenated circulation provided by a living donor).^5^ The long-term results after corrective surgery of TOF in the early years reported a 30-year-survival of 77%.^5^ Since the first surgical correction, both short-term and long-term survival have improved. An early mortality rate of 1.9% to 2.3% has been reported, and the 30-year-survival rate has increased to approximately 90%.^6,7,8^ However, most of these studies were small and single center, focused on postsurgery outcome, and excluded patients who did not undergo surgery.

Tetralogy of Fallot is associated with several genetic conditions. The prevalence of genetic syndromes varies in different studies, from 5% to more than 27%, with 22q11 deletion (DiGeorges syndrome) and trisomy 21 the most frequently reported syndromes. Most of the studies reported an increased risk of mortality associated with the presence of a genetic syndrome.^6,9,10,11^

The aim of this study was to evaluate the nationwide survival in Swedish children with TOF compared with matched controls and note survival changes throughout almost 50 years. This study is, to our knowledge, the first national survey of pediatric mortality in patients with TOF in Sweden.

## Methods

### Data Source and Study Population

We followed the Strengthening the Reporting of Observational Studies in Epidemiology (STROBE) reporting guideline for observational studies. In this nationwide register-based study, we used national medical registers in Sweden to collect data on all patients in Sweden with a recorded diagnosis of TOF, from January 1, 1970, through December 31, 2017. The data were collected from the Swedish National Patient Register^12^ and the Swedish National Cause of Death Register,^13^ both from the Swedish National Board of Health and Welfare. In these registers the diagnoses are coded in accordance with the *International Classification of Diseases, Eighth Revision* (*ICD-8*), *International Classification of Diseases, Ninth Revision* (*ICD-9*), and *International Statistical Classification of Diseases and Related Health Problems, Tenth Revision* (*ICD-10*). All patients with TOF and controls were followed up from the date of birth until event or end of study (December 31, 2017), whichever occurred first. Using the Swedish Total Population Register,^14^ each patient with TOF was initially matched by their year of birth and sex with 10 unique controls (ie, individuals without a diagnosis of TOF). We excluded all patients with TOF and controls who were not born in Sweden and controls who had undergone cardiac surgery for other diagnoses. After the exclusions, the matching was 8.8 controls for each patient with TOF. The study was approved by the Gothenburg Regional Research Ethics Board. In the final data set, all individual national registration numbers were replaced with codes by the Swedish National Board of Health and Welfare in collaboration with Statistics Sweden, which is responsible for official statistics and other government statistics in Sweden. The requirement for informed consent was waived because the study was a large-scale national registry study.

### Definitions

Patients with TOF were identified by using the *ICD* diagnosis codes: Q21.3 (*ICD-10*), 745C (*ICD-9*), and 746.29 (*ICD-8*). Surgery was classified according to Classifications of Operations, 6th edition (Swedish edition, 1970-1996)^15^ and the Classifications of Surgical Procedures, version 1.9 (Swedish edition, 1997-2017).^16^ All-cause death was identified using data from the Swedish National Patient Register and the Swedish National Cause of Death Register. Genetic syndromes associated with TOF were defined as trisomy 21, trisomy 18, trisomy 13, DiGeorge syndrome, CHARGE syndrome (coloboma, heart defects, choanal atresia, growth retardation, genital abnormalities, and ear abnormalities), and VACTERL syndrome (vertebral anomalies, anal atresia, cardiac malformations, tracheoesophageal fistula with esophageal atresia, renal anomalies, and limb anomalies).

### Outcomes

The primary outcome was overall risk of all-cause mortality among patients with TOF overall and patients with TOF who underwent surgery compared with controls. Secondary outcomes were mortality risk by birth period in patients with TOF with and without surgery and in patients with TOF with and without a genetic syndrome.

### Statistical Analysis

Data analysis was performed from September 10 to December 20, 2022. Continuous data are presented as mean (SD) when normally distributed and as median (IQR) when not normally distributed. We used survival analysis techniques for comparing the mortality risk in patients with TOF and controls. The mortality rate was estimated as the number of deaths divided by total follow-up time (person-time) and is reported as per 1000 person-years. We used Kaplan-Meier survival curves with 95% CIs for comparison between patients with TOF and controls, presented as cumulative mortality instead of cumulative survival for better readability. Hazard ratios (HRs) with 95% CIs were calculated using a Cox proportional hazards regression model. All statistical analyses were performed using R, version 3.6.1 (R Foundation for Statistical Computing).^17^ Findings were statistically significant at *P* < .05, with 2-sided unpaired testing.

## Results

### Patients and Controls

In the Swedish national registers, we identified 1848 patients with TOF (784 [42.4%] females, 1064 [57.6%] males) aged 0 to 18 years (mean [SD], 12.4 [6.7] years) and born between 1970 and 2017. We also included a control group of 16 354 individuals (6932 [42.4%] females, 9422 [57.6%] males) who matched the patients with TOF based on year of birth and sex. Other baseline characteristics of the 2 groups are presented in Table 1. Patients and controls were categorized into groups according to birth period by decades from 1970 to 2017. The last birth period comprised only 8 years (2010-2017).

**Table 1.** Study Population Characteristics

Characteristic	Participants, No. (%)	
	TOF (n = 1848)	Control (n = 16 354)
Sex		
Female		
Overall	784 (42.4)	6 932 (42.4)
1970-1979	132 (42.0)	1120 (44.1)
1980-1989	149 (41.3)	1156 (41.9)
1990-1999	200 (45.4)	1725 (45.0)
2000-2009	167 (41.1)	1609 (41.0)
2010-2017	136 (41.7)	1322 (40.0)
Male		
Overall	1064 (57.6)	9422 (57.6)
1970-1979	182 (58.0)	1420 (55.9)
1980-1989	212 (58.7)	1601 (58.1)
1990-1999	241 (54.6)	2111 (55.0)
2000-2009	239 (58.9)	2311 (59.0)
2010-2017	190 (58.3)	1979 (60.0)
Cardiac surgery^a^	1527 (82.6)	NA
Genetic syndrome	294 (15.9)	15 (0.1)
Deaths	286 (15.5)	91 (0.6)
Follow-up, mean (SD), y	12.4 (6.7)	14.0 (5.5)

Abbreviations: NA, not applicable; TOF, tetralogy of Fallot.

^a^ Patients with TOF who underwent cardiac surgery before age 18 years.

### All-Cause Mortality

In our cohort including 1848 patients with TOF, we observed increased survival from 68.5% to 96.0% in children born in the 1970s compared with those born in the 2010s. The risk of mortality associated with congenital cardiac surgery decreased substantially but was 11 times higher than the risk in controls. The mean (SD) follow-up time was 12.4 (6.7) years in the TOF group and 14.0 (5.5) years in the control group. At the end of follow-up, 286 patients (15.5%) with TOF and 91 controls had died, which renders the overall mortality risk almost 30 times higher in the TOF group (HR, 29.8; 95% CI, 23.6-37.8) compared with the control group (Table 2). The number of patients with TOF who underwent surgery was 1527 (897 [58.7%] males, 630 [41.3%] females) of whom 21 patients (1.4%) had pulmonary atresia. In patients who underwent surgery, 154 (10.1%) died (Table 3) during a mean (SD) follow-up time of 13.6 (5.7) years, with a mortality risk of 21.9 (95% CI, 16.2-29.7) compared with matched controls. The overall cumulative mortality in patients with TOF and controls is illustrated in eFigure 1A in Supplement 1. The cumulative mortality in patients with TOF and matched controls differentiated by birth periods is illustrated in eFigure 1B in Supplement 1. There was no significant difference in mortality between male and female patients with TOF (eFigure 1C in Supplement 1).

**Table 2.** Risk of All-Cause Mortality in All Patients With TOF Compared With Controls

Variable	TOF		Controls		HR for mortality (95% CI)^a^
	Deaths, No. (%)	Mortality rate per 1000 person-years (95% CI)	Deaths, No. (%)	Mortality rate per 1000 person-years (95% CI)	
Total	286 (15.5)	125.0 (110.9-140.3)	91 (0.6)	4.0 (3.2-4.9)	29.8 (23.6-37.8)
Birth period					
1970-1979	99 (31.5)	233.9 (190.1-284.7)	23 (0.9)	5.1 (3.2-7.6)	40.7 (25.9-64.1)
1980-1989	84 (23.3)	161.2 (128.6-199.6)	21 (0.8)	4.3 (2.6-6.5)	34.4 (21.3-55.4)
1990-1999	64 (14.5)	92.1 (71.0-117.7)	24 (0.6)	3.5 (2.2-5.2)	24.8 (15.5-39.7)
2000-2009	26 (6.4)	51.3 (33.5-75.2)	15 (0.4)	2.9 (1.6-4.9)	17.2 (9.1-32.4)
2010-2017	13 (4.0)	90.9 (48.4-155.4)	8 (0.2)	5.3 (2.3-10.5)	16.7 (6.9-40.3)
Sex					
Male	155 (54.2)	116.7(99.1-136.6)	67 (73.6)	51.4 (39.8-65.2)	21.8 (16.3-29.0)
Female	131 (45.8)	136.3(114.0-161.8)	24 (26.4)	24.3 (15.6-36.1)	52.3 (33.9-80.9)

Abbreviations: HR, hazard ratio; TOF, tetralogy of Fallot.

^a^ HR compared with controls from the same birth period and same sex.

**Table 3.** Risk of All-Cause Mortality in Patients With TOF Who Underwent Surgery Compared With Controls

Variable	TOF with surgery		Controls		HR for mortality (95% CI)^a^
	Deaths, No. (%)	Mortality rate per 1000 person-years (95% CI)	Deaths, No. (%)	Mortality rate per 1000 person-years (95% CI)	
Total	154 (10.1)	7.82 (6.64-9.16)	57 (0.47)	0.35 (0.26-0.45)	21.9 (16.2-29.7)
Birth period					
1970-1979	62 (27.3)	19.23 (14.7-24.6)	12 (0.8%)	0.4 (0.22-0.75)	40.6 (21.9-75.4)
1980-1989	52 (18.0)	11.66 (8.7-15.3)	13 (0.7%)	0.4 (0.20-0.65)	28.7 (15.6-52.7)
1990-1999	24 (6.9)	4.03 (2.6-6.0)	15 (0.5%)	0.3 (0.17-0.50)	12.8 (6.7-24.5)
2000-2009	10 (2.8)	2.14 (1.0-3.9)	12 (0.4%)	0.3 (0.16-0.53)	7.0 (3.0-16.3)
2010-2017	6 (2.0)	4.36 (1.6-9.5)	5 (0.2%)	0.4 (0.13-0.92)	11.1 (3.4-36.4)
Sex					
Male	94 (10.5)	81.6 (66-99.9)	43 (0.6%)	4.52 (3.3-6.1)	17.6 (12.3-25.3)
Female	60 (9.5)	73.5 (56.1-94.5)	14 (0.3%)	2.05 (1.1-3.4)	35.2 (19.6-62.9)

Abbreviations: HR, hazard ratio; TOF, tetralogy of Fallot.

^a^ Hazard ratio compared with controls from the same birth period and same sex.

### Mortality Rates and Mortality Risks

The overall mortality rate was 125.0 per 1000 person-years in the TOF group and 4.0 per 1 000 person-years in the control group. The lowest mortality rate in patients with TOF was observed in the birth period 2000-2009, with a mortality rate of 51.3 (95% CI, 33.5-75.2) per 1000 person-years (Table 2). In the same birth period, the mortality rate among controls was 2.9 (95% CI, 1.6-4.9) per 1000 person-years. The mortality risk in the last birth period analyzed (ie, 2010-2017) was almost 17 times higher in patients with TOF compared with controls (HR, 16.7; 95% CI, 6.9-40.3). Nevertheless, this risk is substantially lower than in the first birth period (ie, 1970-1979), in which the mortality risk was more than 40 times higher (HR, 40.7; 95% CI, 25.9-64.1). The corresponding decrease in mortality risk for patients with TOF who underwent surgery was 40.6 (95% CI, 21.9-75.4) in the birth period 1970 to 1979 and 11.1 (95% CI, 3.4-36.4) in the birth period 2010 to 2017 (Table 3). The survival rate, which was 68.5% in the birth period 1970 to 1979, increased to 93.6% in the birth period 2000-2009, and to 96.0% in the birth period 2010 to 2017 while the survival rate in children who underwent cardiac surgery increased to 98.0%.

### Mortality Risks With Surgery

**Figure 1.**
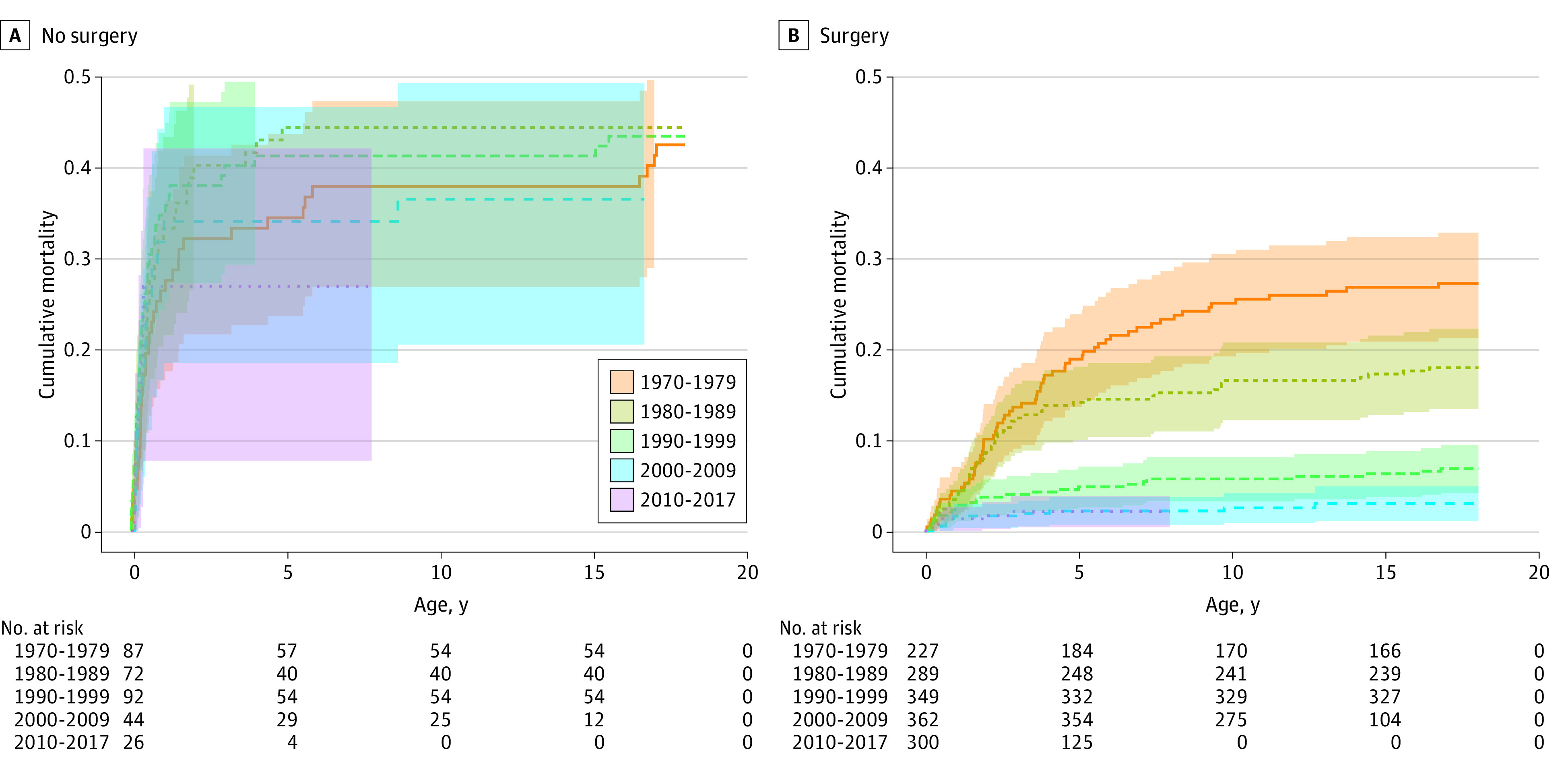
Cumulative Mortality in Patients With Tetralogy of Fallot With and Without Congenital Cardiac Surgery Patients with tetralogy of Fallot who did not undergo cardiac surgery (A) and those who underwent cardiac surgery (B) from birth to age 18 years.

During the study period, a total of 1527 patients with TOF underwent cardiac surgery between birth and age 18 years. The number of patients with TOF who had cardiac surgery during childhood increased over the time of the study. In the first birth period, 72.3% of the patients had cardiac surgery compared with 92.0% of the patients in the last birth period. The mortality rate among patients who had surgery decreased during the study period, from 27.3% in 1970 to 1979 to 2.0% in 2010 to 2017. For an infant born with TOF and no identified genetic syndrome who underwent cardiac surgery the change in survival, compared with controls, is illustrated in Figure 1. There was no clear improvement over time in patients with TOF without surgery, while in those who had undergone surgery, the survival probability increased with the more recent birth periods. The overall cumulative mortality of patients with TOF with and without surgery is shown in eFigure 2A in Supplement 1. The HR for mortality in patients with TOF who underwent cardiac surgery in all birth periods was 0.19 (95% CI, 0.15-0.24) (eTable 3 in Supplement 1). The HRs for mortality have decreased with later birth years, and in the last study cohort (2010-2017) the risk of mortality was 20 times lower in the group of patients with TOF who had cardiac surgery compared with the group of patients who did not have surgery (HR, 0.05; 95% CI, 0.02-0.16) (eTable 3 in Supplement 1).

### The Role of Genetic Syndromes

**Figure 2.**
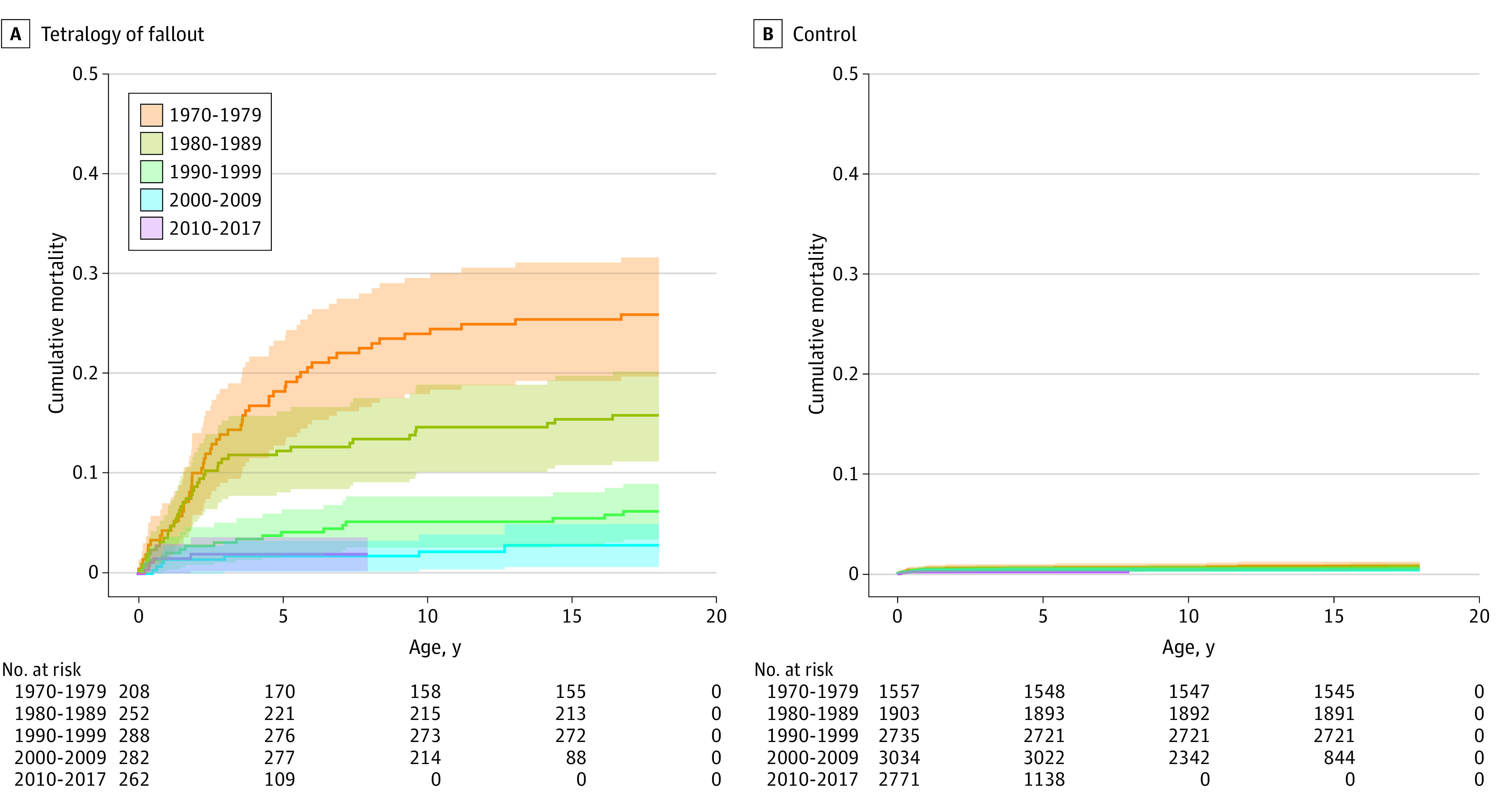
Cumulative Mortality in Patients With Tetralogy of Fallot Without Genetic Syndromes Who Underwent Congenital Surgery Patients with tetralogy of Fallot without genetic syndromes who underwent congenital surgery (A) and matched controls (B) from birth to age 18 years.

Figure 2 illustrates the cumulative mortality in patients with TOF without genetic syndromes who underwent surgery and matched controls from birth to age 18 years differentiated by birth periods. In the population of patients with TOF, 294 children (15.9%) had one of the genetic syndromes depicted in eTable 1 in Supplement 1, compared with 15 children (0.1%) in the control group (Table 1). The genetic syndromes included in our study were trisomy 21, trisomy 18, trisomy 13, DiGeorge syndrome, VACTERL, and CHARGE. The most common genetic syndrome in both study groups was trisomy 21. Among those who died in the group of patients with TOF during follow-up, there was a higher percentage of children with genetic syndromes (25.2%) compared with the entire group of TOF patients (1970-1979 [34.6%], 1980-1989 [29.4%], 1990-1999 [22.4%], 2000-2009 [9.1%], 2010-2017 [4.5%]) (eTable 2 in Supplement 1). The difference in overall cumulative mortality in patients with TOF with and without genetic syndromes is illustrated in eFigure 2B in Supplement 1, illustrating a clear advantage in the group without a genetic syndrome. The cumulative mortality of patients with TOF with a genetic syndrome over time differentiated by birth period is illustrated in eFigure 2C in Supplement 1, suggesting an improvement in survival in this group of patients. The overall cumulative mortality in patients with TOF without a genetic syndrome compared with controls is illustrated in eFigure 2D in Supplement 1.

## Discussion

Our analysis of Swedish nationwide registry data from 1970 to 2017 noted a marked total reduction in mortality among pediatric patients with TOF during this time. The survival rate, which was 68.5% in the birth period 1970 to 1979, increased to 93.6% in the birth period 2000 to 2009, and to 96.0% in the birth period 2010 to 2017, and the survival rate in children who underwent cardiac surgery increased to 98.0%. eFigure 3 in Supplement 1 illustrates the change of HRs for mortality in patients with TOF during almost 50 years with 1970 as the reference. The gradual decrease in mortality risk suggests a continuous improvement in the care of these patients rather than a few sudden sizable changes. Most likely a combination of advances in the care of this group of patients over time explains the results, for example, improvements in TOF identification prenatally, surgical techniques, perioperative care, and advancement during the long-term follow-up.

However, there remains an increased mortality risk among patients with TOF compared with matched controls. The relative risk of death during childhood is approximately 17 times higher in children with TOF, even after 2000. By differentiating the TOF population based on the occurrence of cardiac surgery, we could, according to HRs, ascertain a lower risk of mortality in individuals who undergo congenital cardiac surgery. The fact that among the patients who died during childhood, 50% had not undergone cardiac surgery supports the notion. However, approximately 10% of children with TOF remained without corrective surgery after 2000.

Large and nationwide studies indicating decreases in mortality risk over time among children and young adults with CHDs have been published.^18,19,20,21,22^ Two of these studies by Mandalenakis et al^18,19^ have included both patients with and without surgery and cover a total cohort of patients with CHD in Sweden. The studies describe survival trends in children and young adults with CHD compared with matched controls, using Swedish health registers. In the study published in 2020,^18^ the authors reported survival rates increasing from 70% for children born in the 1980s to more than 90% in those born in the 2010s with conotruncal heart defects including TOF. Compared with matched controls, patients with conotruncal heart defects still had an almost 50 times greater mortality risk.

The other 3 studies^20,21,22^ focused on reporting trends in survival in patients with CHD after congenital cardiac surgery and/or cardiac interventions. They all noted a distinct improvement in survival over time in patients with all types of CHD, including a subset of patients with TOF and congenital cardiac surgery, congruent with our results. Larsen et al^20^ reported, in a Danish nationwide study, 10-year freedom of death among patients with TOF who underwent surgery of 61% in 1977 to 1989 and 95% in 2003 to 2015. A Norwegian study of postoperative survival in patients with TOF reported a cumulative survival of 86.3% until patients were aged 16 years in 1970 to 1989 and 94.3% in 1990 to 2011.^21^ Raissadati et al^22^ reported, in a 6-decade study in Finland, a 22-year survival rate postsurgery in patients with TOF that improved from 87% in 1953 to 1989 up to 90% in 1990 to 2009, with an HR of 0.52 for death in 1990 to 2009 (95% CI, 0.28-0.94). The same study also reported a significant decrease in early mortality (up to 30 postoperative days) from 9% in 1953 to 1989 to 1% in 2000 to 2009. A large US study using data from the Pediatric Cardiac Care Consortium,^23^ found an increased risk of long-term mortality after surgery for CHDs compared with the general population. When specifically studying survival trends in patients with TOF, the investigators noted an improvement from the first study period (1982-1992), with a 15-year survival estimate of 91.4%, to the latest period (1998-2003), with a 15-year survival estimate of 93.7%.

The improvement in survival rates in our study is consistent with the findings in other studies focusing on TOF. Many of the specific TOF studies are single-center studies concentrating on postsurgical short- and long-term outcomes.^24,25^ Hickey et al^24^ reported an improvement in early surgical mortality among 1181 patients with TOF during 4 study decades (1960-1998) and an overall mean (SD) survival rate during childhood of 85% (1%). Lindberg et al^25^ noted an improvement in early mortality (up to 30 postsurgery days) from 40.1% in 1953 to 1971 to 0.6% in 2000 to 2008.

Regarding patients with TOF who have genetic conditions, our results suggest a lower survival rate in this patient group compared with patients with TOF without genetic conditions, in line with earlier studies.^9,10,11^ Blais et al^9^ reported a prevalence of a genetic syndrome of 17.5% (trisomy 21, 22q11-deletion and other syndromes) in their study population of patients with TOF in Canada and a significantly lower 30-year survival among patients with trisomy 21 (84.6%) and other syndromes (77.2%) compared patients with TOF without syndromes (94.7%). In an Italian study,^10^ a genetic defect was found in up to 27.8% of the patients with TOF, with 22q11 deletion, trisomy 21, VACTERL, and CHARGE being the most frequent ones. The patients with a syndrome had a significantly lower mean (SD) survival rate 10.4 years after surgical correction (84.3% [4.2%]) compared with patients without a syndrome (94.1% [2.3%]) (*P* < .001). In a study by Smith et al,^11^ using data from the Pediatric Cardiac Care Consortium in the US, the authors found a prevalence of genetic conditions of 11% (trisomies, DiGeorge syndrome, and other conditions) and significantly decreased survival among these patients compared with patients with TOF without a genetic condition (HR, 3.64; 95% CI, 2.05-6.47).

### Strengths and Limitations

In our study, there are some factors that strengthen it, the main one being the large number of included patients and its coverage of nearly 5 decades (48 years). Furthermore, the data in our study were extracted from nationwide registers with good quality enabling us to achieve almost complete follow-up. Since our aim was to thoroughly describe the entire cohort of patients with TOF and their survival over time, we chose to include all patients with a diagnosis of TOF and not just those who had undergone congenital cardiac surgery. Much of the literature on children and adults with TOF has a focus on postsurgical survival and omits the group of patients without congenital cardiac surgery. Our study suggests the paramount importance of congenital factors in regard to survival probability. Our study also included a control population matched on birth year and sex enabling us to compare survival not only within the group of patients with TOF but also with the general population.

Our study has limitations. First, the follow-up period for patients born after 2000 (ie, the 2 last birth periods) was shorter than for patients born before 2000. Patients born after 2000 were not aged at least 18 years before the end of the study in December 2017; therefore, the improved survival in these patients may not be quite accurate. Despite this, based on the data we can suggest that most of the diminishing difference in mortality between patients with TOF and controls had already occurred in the beginning of the new millennium.

Second, the study was based on data from registers and we did not have any clinical data available. Therefore, we were not able to categorize the patients with TOF by the severity of their anatomic defects, which could have been interesting because of the heterogenicity of the diagnosis itself. As in all data from registers there is a risk of misclassification of diagnoses and procedures. To our knowledge, there is no publication on the formal validations of the diagnostic codes of CHDs in the Swedish national health registers; however, the Swedish National Inpatient register has been reported to have high validity for many diagnoses.^26^ Owing to the long span of the study period, we used 3 different versions of *ICD* codes (*ICD-8* from 1970-1986, *ICD-9* from 1987-1996, and *ICD-10* from 1997 and thereafter), which might affect the comparability of the patients with TOF. Third, our study has the limitation that genetic syndromes may have been less well captured in the early years of our investigation, and there are other genetic conditions that may not have been captured but could contribute to TOF and its outcome.

## Conclusion

In this study, we observed a significant reduction in absolute and relative mortality among patients with TOF from 1970 to 2017. However, there still exists a considerable increased risk of in mortality in patients with TOF compared with age- and sex-matched controls. The remaining challenge is to identify the routes for further survival improvements for pediatric patients with TOF by noting the predictors of good and poor outcomes and determining the modifiable factors for further outcome improvements.

## Notes

Supplement 1. **eTable 1.** Genetic Syndromes Associated With TOF and Their Occurrence Rate in the Study Population **eTable 2.** Number and Characteristics of TOF Patients and Controls Who Died **eTable 3.** Cardiac Surgery and Risk of Mortality **eFigure 1.** The Cumulative Mortality in Patients vs Controls **eFigure 2.** The Cumulative Mortality in Patients With TOF **eFigure 3.** The Overall Hazard Ratios for Mortality

Supplement 2.
Data Sharing Statement
