# Supplementary material for: Survival Trends in Children With Tetralogy of Fallot in Sweden From 1970 to 2017
Source: JAMA Netw Open. 2023 May 22;6(5):e2314504. doi: 10.1001/jamanetworkopen.2023.14504 (PMC10203890; doi:10.1001/jamanetworkopen.2023.14504)
Supplement: Supplement 1. — eTable 1. Genetic Syndromes Associated With TOF and Their Occurrence Rate in the Study Population eTable 2. Number and Characteristics of TOF Patients and Controls Who Died eTable 3. Cardiac Surgery and Risk of Mortality eFigure 1. The Cumulative Mortality in Patients vs Controls eFigure 2. The Cumulative Mortality in Patients With TOF eFigure 3. The Overall Hazard Ratios for Mortality [file jamanetwopen-e2314504-s001.pdf]

Supplementary Online Content

Persson J, Gyllencreutz Castellheim A, Dellborg M, et al. Survival trends in children with tetralogy of Fallot in Sweden from 1970 to 2017. *JAMA Netw Open*. 2023;6(5):e2314504. doi:10.1001/jamanetworkopen.2023.14504

eTable 1. Genetic Syndromes Associated With TOF and Their Occurrence Rate in the Study Population

eTable 2. Number and Characteristics of TOF Patients and Controls Who Died

eTable 3. Cardiac Surgery and Risk of Mortality

eFigure 1. The Cumulative Mortality in Patients vs Controls

eFigure 2. The Cumulative Mortality in Patients With TOF

eFigure 3. The Overall Hazard Ratios for Mortality

This supplementary material has been provided by the authors to give readers additional information about their work.

© 2023 Persson J et al. *JAMA Network Open.*

eTable 1: Genetic syndromes associated with TOF and their occurrence rate in the study population

Genetic syndrome	ICD code [a]	TOF-patients, n (%)	Controls, n (%)	p-value
Trisomy 21	ICD-10: Q90.0, Q90.1, Q90.2, Q90.9<br>ICD-9: 758A/758.0 [b]<br>ICD-8: 759.30/759.3 [b]	167 (9.0)	9 (0.1)	<0.001
Trisomy 18	ICD-10: Q91.0, Q91.1, Q91.2, Q91.3<br>ICD-9: 758C/758.2 [b]<br>ICD-8: 759.40/759.4 [b]	24 (1.3)	0 (0.0)	<0.001
Trisomy 13	ICD-10: Q91.4, Q91.5, Q91.6, Q91.7<br>ICD-9: 758B/758.1 [b]<br>ICD-8: 759.41/759.4 [b]	11 (0.6)	0 (0.0)	<0.001
DiGeorge syndrome	ICD-10: D82.1<br>ICD-9: 279L/279.1 [b]<br>ICD-8: -	75 (4.1)	0 (0.0)	<0.001
CHARGE	ICD-10: Q87.8<br>ICD-9: -<br>ICD-8: -	23 (1.2)	6 (0.0)	<0.001
VACTERL	ICD-10: Q87.2W<br>ICD-9: -<br>ICD-8: -	3 (0.2)	0 (0.0)	<0.001

ICD, International Classification of Diseases; CHARGE, Coloboma of the eye, Heart defects, Atresia of the choanae, Restriction of Growth and development, and Ear abnormalities and deafness; VACTERL, vertebral defects, anal atresia, cardiac defects, tracheo-esophageal fistula, renal anomalies, and limb abnormalities.

[a] ICD-8 1969-1986, ICD-9 1987-1996, ICD-10 1997-.

[b] ICD-codes when used in the National Patient Register/when used in the National Cause of Death Register.

The table demonstrates associated genetic syndromes with TOF. ICD-codes 8, 9 and 10 for these syndromes are shown. The table illustrates the occurrence rate of the genetic syndromes among the TOF patients and the controls.

© 2023 Persson J et al. *JAMA Network Open.*

eTable 2. Number and characteristics of TOF patients and controls who died

**Characteristic**	**TOF patients (n=286)** n (%)	**Controls (n=91)** n (%)
1970–1979	99 (34.6)	23 (25.3)
1980–1989	84 (29.4)	21 (23.1)
1990–1999	64 (22.4)	24 (26.4)
2000–2009	26 (9.1)	15 (16.5)
2010–2017	13 (4.5)	8 (8.8)
Male	155 (54.2)	67 (73.6)
Congenital cardiac surgery [a]	154 (53.8)	0 (0.0)
Genetic syndrome	72 (25.2)	0 (0.0)
Mean follow-up	year (SD) 2.8 ± 4.0	year (SD) 1.7 ± 3.8

SD, standard deviation; [a] TOF patients with congenital cardiac surgery before 18 years of age.
The table demonstrates characteristics of TOF patients and matched controls who died during follow-up.

© 2023 Persson J et al. *JAMA Network Open.*

eTable 3. Cardiac surgery and risk of mortality

Birth periods years	Cardiac surgery *n* (%)	Number of deaths n (%)	Hazard ratiions (HR) HR (95% CI for mortality) [a]
1970-1979	227 (72.3)	62 (27.3)	0.52 (0.35-0.79)
1980-1989	289 (80.1)	52 (18.0)	0.30 (0.19-0.46)
1990-1999	349 (79.1)	24 (6.9)	0.12 (0.07-0.20)
2000-2009	362 (89.2)	10 (2.8)	0.06 (0.03-0.13)
2010-2017	300 (92.0)	6 (2.0)	0.05 (0.02-0.16)
All birth periods	1527 (82.6)	154 (10.1)	0.19 (0.15-0.24)

CI, confidence interval
[a] Compared with TOF patients and no cardiac surgery before 18 years of age
The table demonstrates risk of mortality in TOF patients who had congenital cardiac surgery before the age of 18 years differentiated by birth period.

© 2023 Persson J et al. *JAMA Network Open.*

eFigure 1. The Cumulative Mortality in Patients vs Controls

eFigure 1A: The overall cumulative mortality in TOF patients and controls

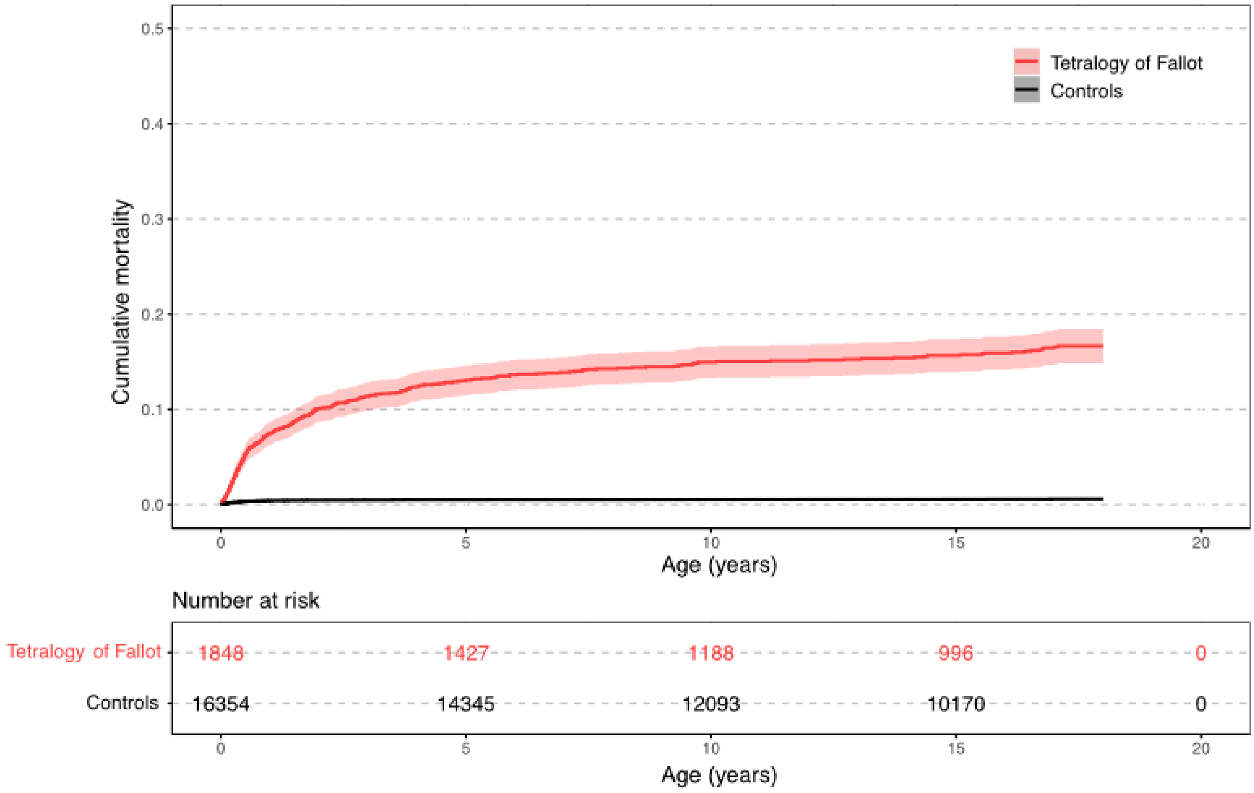

The figure illustrates the overall cumulative mortality in TOF patients and matched controls from birth to 18 years.

© 2023 Persson J et al. *JAMA Network Open.*

eFigure 1B: The cumulative mortality differentiated by birth periods

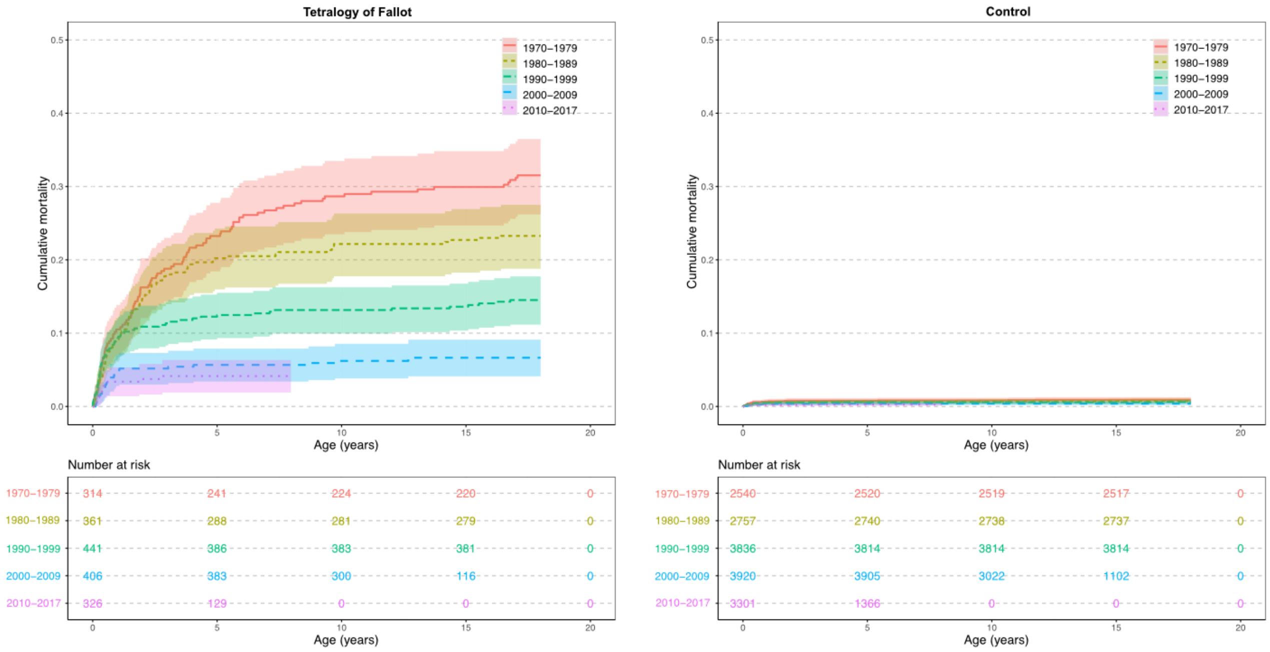

The figure illustrates cumulative mortality in TOF patients (left) and matched controls (right) from birth to 18 years differentiated by birth periods.

© 2023 Persson J et al. *JAMA Network Open.*

eFigure 1C: The overall cumulative mortality by sex

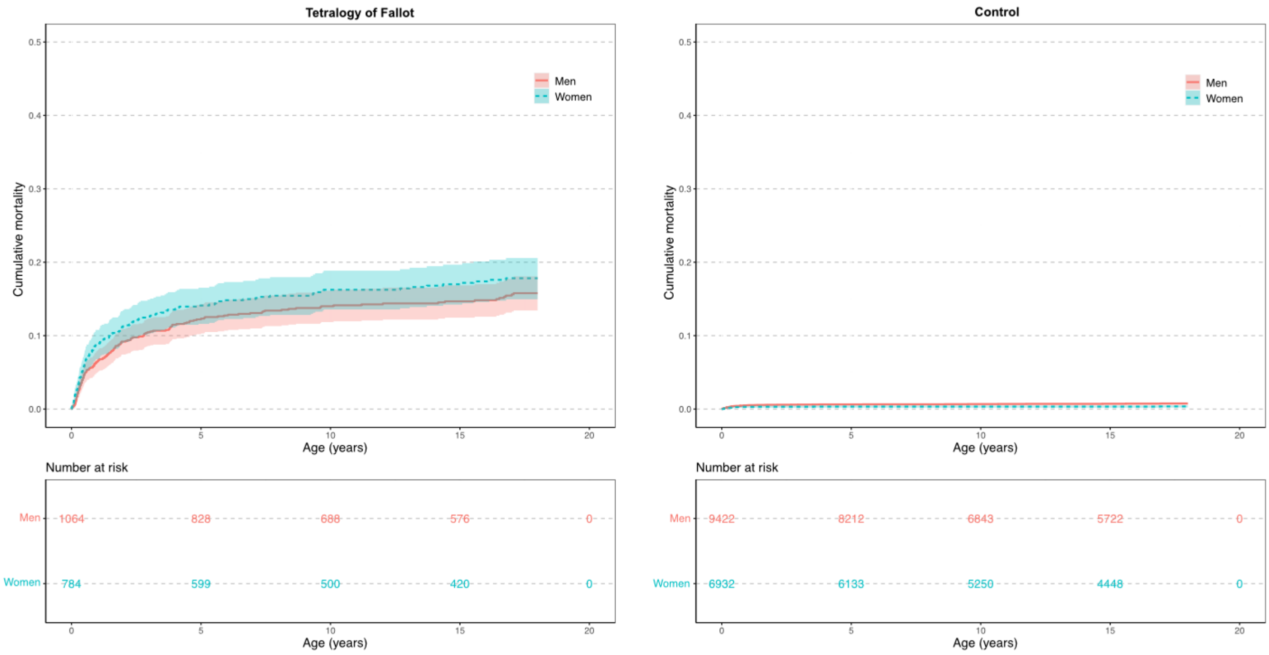

The figure illustrates overall cumulative mortality in TOF patients (left) and matched controls (right) from birth to 18 years differentiated by sex.

© 2023 Persson J et al. *JAMA Network Open.*

eFigure 2. The Cumulative Mortality in Patients With TOF

eFigure 2A: The overall cumulative mortality by presence or absence of surgery

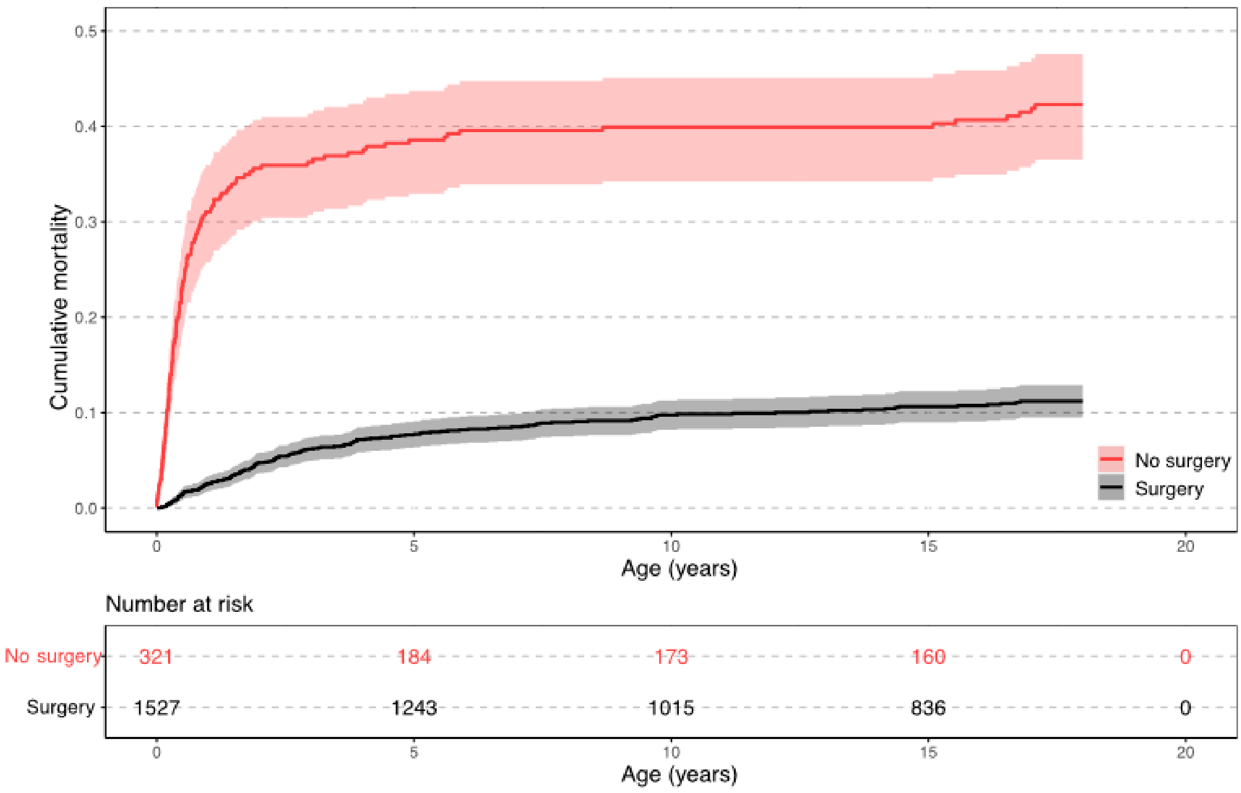

The figure illustrates overall cumulative mortality in TOF patients from birth to 18 years differentiated by presence or absence of congenital cardiac surgery during childhood.

© 2023 Persson J et al. *JAMA Network Open.*

eFigure 2B: The overall cumulative mortality by presence or absence of a genetic syndrome

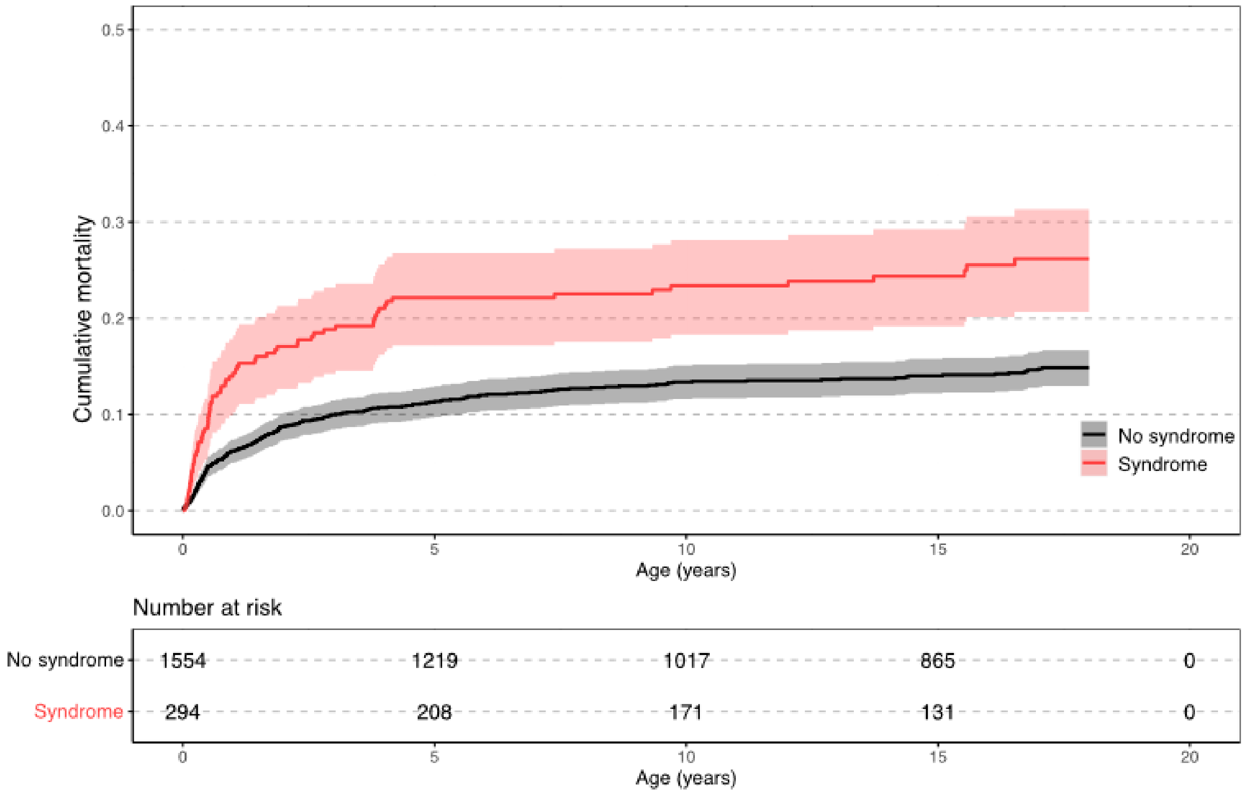

The figure illustrates overall cumulative mortality in TOF patients from birth to 18 years differentiated by presence or absence of a genetic syndrome.

© 2023 Persson J et al. *JAMA Network Open.*

eFigure 2C: The cumulative mortality by presence or absence of a genetic syndrome and birth periods

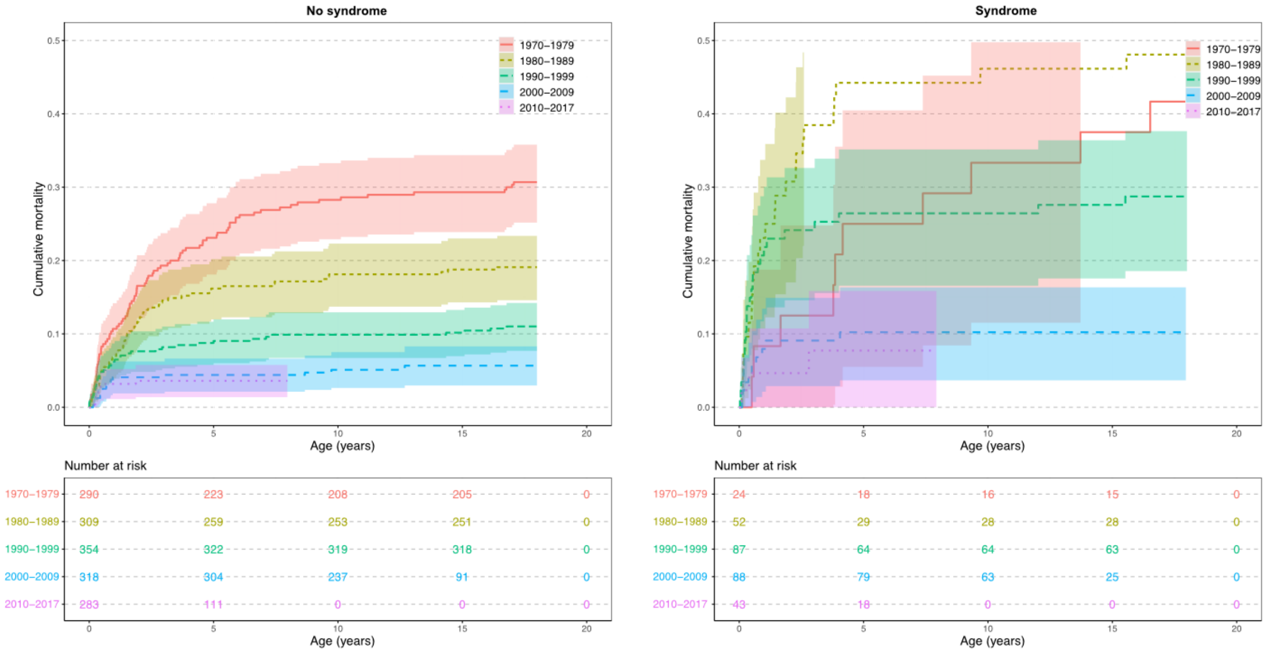

The figure illustrates cumulative mortality in TOF patients without (left) or with (right) a genetic syndrome from birth to 18 years differentiated by birth periods.

© 2023 Persson J et al. *JAMA Network Open.*

eFigure 2D: The overall cumulative mortality without syndrome

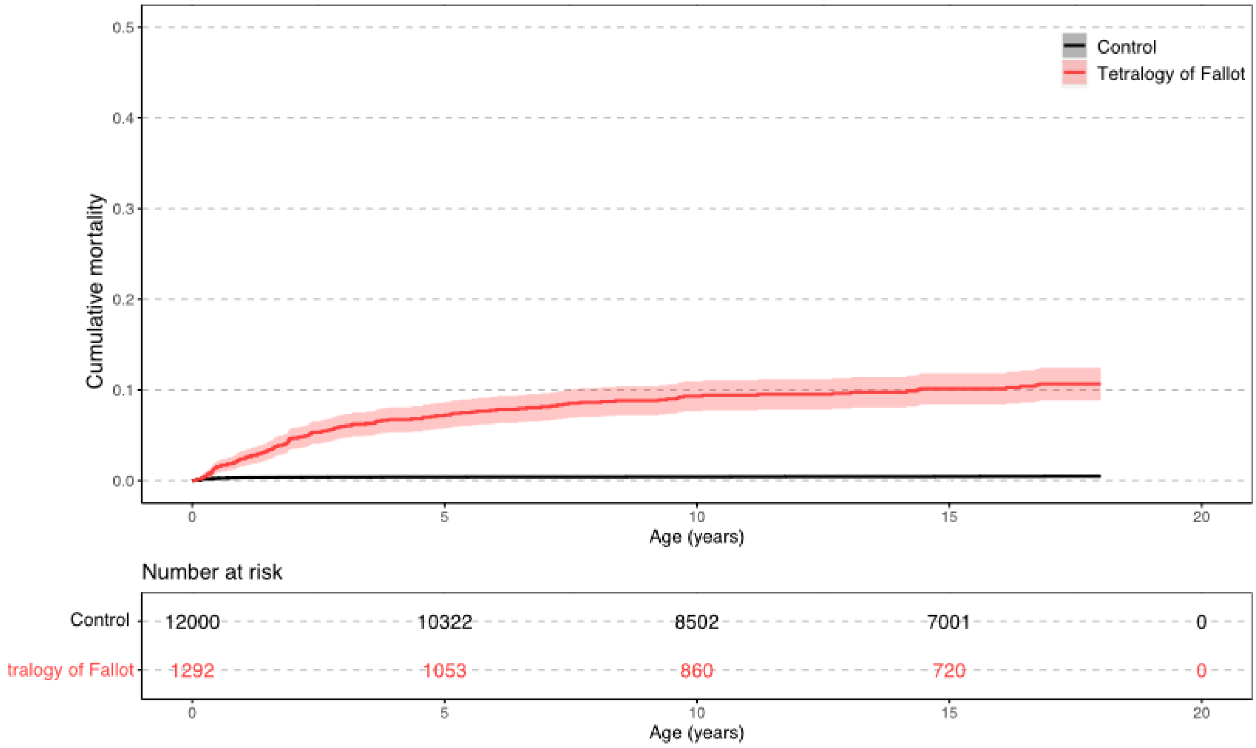

The figure illustrates overall cumulative mortality of TOF patients without syndrome compared with controls.

© 2023 Persson J et al. *JAMA Network Open.*

eFigure 3: The overall hazard ratios for mortality

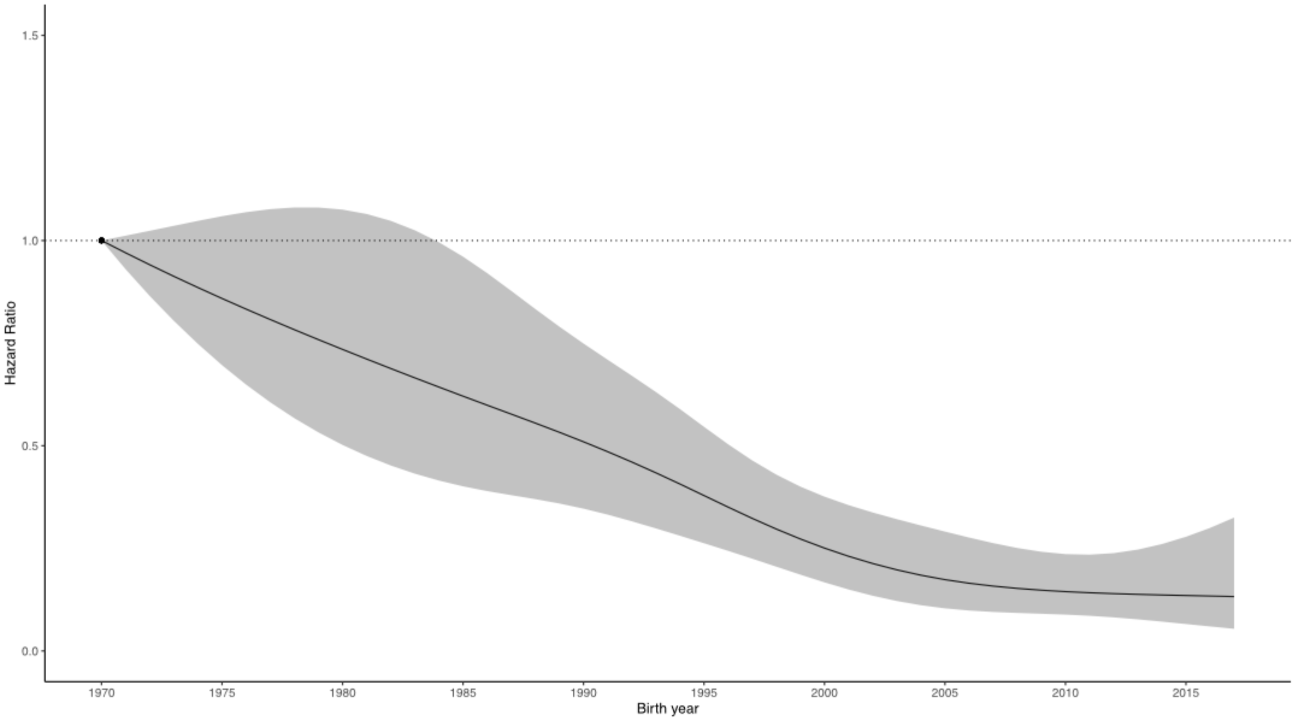

The figure illustrates overall hazard ratios for mortality in TOF patients aged 0 to 18 from 1970 to 2017, with the year 1970 as reference

© 2023 Persson J et al. *JAMA Network Open.*
