# Supplementary material for: Survival Trends in Children With Tetralogy of Fallot in Sweden From 1970 to 2017
Source: JAMA Netw Open. 2023 May 22;6(5):e2314504. doi: 10.1001/jamanetworkopen.2023.14504 (PMC10203890; doi:10.1001/jamanetworkopen.2023.14504)
Supplement: Supplement 2. — Data Sharing Statement [file jamanetwopen-e2314504-s002.pdf]

Data Sharing Statement

Persson. Survival Trends in Children With Tetralogy of Fallot in Sweden From 1970 to 2017. *JAMA Netw Open*. Published May 22, 2023. doi:10.1001/jamanetworkopen.2023.14504

Data
Data available: No
